# Supplementary material for: Exploring family functioning and ‐ hardiness in families’ experiencing adult intensive care – A cross-sectional study
Source: PLoS One. 2023 Jul 6;18(7):e0288149. doi: 10.1371/journal.pone.0288149 (PMC10325063; doi:10.1371/journal.pone.0288149)
Supplement: Attachment — Submitted filename: Addressing the comments 230105.rtf [file pone.0288149.s002.rtf]

Addressing the comments:
Thank you for reviewing this manuscript, the feedback of every subject was very clear and I hope you find the adjustments to be appropriate.
subject	Journal requirements 	answer	
1. Please ensure that your manuscript meets PLOS ONE's style requirements, including those for file naming.		Manuscript adjusted according the requirements	
2. We note that the grant information you provided in the 'Funding Information' and 'Financial Disclosure' sections do not match. When you resubmit, please ensure that you provide the correct grant numbers for the awards you received for your study in the 'Funding Information' section.		The grant number is Health Research Council in the South-East of Sweden (FORSS 466311).” In the Funding information section	
3. Thank you for stating the following financial disclosure: “The present study is supported, in part, by a grant from the Health Research Council in the South-East of Sweden (FORSS 466311).” Please state what role the funders took in the study.  If the funders had no role, please state: "The funders had no role in study design, data collection and analysis, decision to publish, or preparation of the manuscript." If this statement is not correct you must amend it as needed. Please include this amended Role of Funder statement in your cover letter; we will change the online submission form on your behalf.		 Thank you,  the statement “The funders had no role in study design, data collection and analysis, decision to publish, or preparation of the manuscript.” will be added in the cover letter	
4. We note that you have stated that you will provide repository information for your data at acceptance. Should your manuscript be accepted for publication, we will hold it until you provide the relevant accession numbers or DOIs necessary to access your data. If you wish to make changes to your Data Availability statement, please describe these changes in your cover letter and we will update your Data Availability statement to reflect the information you provide.		Sorry wrong of me. Data are stored safe without any personal information.	
5. Please include your tables as part of your main manuscript and remove the individual files. Please note that supplementary tables (should remain/ be uploaded) as separate "supporting information" files		Thank you for the information, the manuscript has been adjusted	
SUBJECT	REVIEWER 1 COMMENTS	ANSWER	
SERIOUS CONCERNS			
Statistics:	The GFS score is the mean of all 12 items, with a range between 1 and 4, correct? If the mean GFS was 1.5 (0.6), the result may not be normal distributed. Please check your data for distribution. In general, normal distributed data are reported as mean and standard deviation, and for metrical data tests such as t-tests etc can be used. Non-normal distributed data are reported as median and Interquartil Range (IQR), and other tests should be used for metrical data, such as Mann-Whitney U Test or other. Please, check the data for distribution and use appropriate tests. Please add this information to section data analysis, and – if needed- revise the results.	The GF is a summative scale, where the total score is the mean of all items, divided in 12. Negative items are transformed before calculating the GFS score.
See table 1 and 2.	
	Same to FHI.	See Table 1 and 2	
Tables:	where are the tables???	Now included in the manuscript	
MINOR CONCERNS			
Section abstract is well written.	Interestingly, it contains relevant information without reporting numbers (except for patients). Unusual, but fine.		
	Page 3, Line 49: The references are numbered, but “SIR, 2022” not. Please check.	Sorry missed this has now been adjusted	
Section background is fine.	Main concepts are defined and referenced.		
P4, L88	I wonder if the setting is correct? Maybe you recruited patients in this setting, but patients and families were surveyed at home, right?	This is correct. The former patients and their family were recruited at home after being cared for at four general ICUs in two regional
P4, L89-90	
P5, L93	to be clear “the participants” were former ICU patients? Patients were not recruited while being cared on an ICU, following the inclusion criteria, but recruited from a data base of former patients. If correct, you should revise add a term similar to “from a database including former patients”, or else.
Very good: these persons are not patients anymore, and you use the term “participants”. Excellent!	ICU administrators that listed from a database including former patients, participants eligible for inclusion. The inclusion criteria were: alive former ICU patients
P5, L95-96	
P5, L95	is that all inclusion criteria? Alive, ≥96h ICU, and ≥19y? What about cognitive capability to fill in a questionnaire, understand language, and others? Or having a family? What happened to those without family?	The inclusion criteria was not adjusted for cognitive capability, understanding Swedish or former patients having family. The family was defined as the persons the family considered being family members, regardless of living in the same household or not. Those former patients not fulfilling these conclusions were probably not participating  	
P5, L99	why 390 patients (patients or participants?)?	Adjusted to participants	
P5, L103	okay, to be clear: when persons (humans) are treated in hospital or go to the GP, they are becoming “patients”, including contracts, dependency, treatments etc etc etc. But after discharge, the contract is finished, and they are … persons, participants, citizens, men, women, humans again. With respect to the dignity of the persons and their families, and the non-ICU setting, these attributes should be clearly used. Please revise throughout the manuscript.	Thank you adjusted in the manuscript	
Section methods	I am impressed by the clear reports of the questionnaires. Well done.		
P7, L160	“104”? What does it mean? Reference?	The calculation is based on a medium power size, 104 participants were needed.
ES = 0.6, á = 0:05; 1 − â = 0.8
	
P10, L228	in the first paragraph in section discussion, the design and main results are reported, without adding new results, or references. Just a short overview for fast readers. Please revise.	The first section has been removed	
Limits	you may shorten the section limitations to the core meaning.	Thank you I have shorten the text	
Conclusions	conclusions are based on methods, results, discussion, and limitations. Conclusions are generalizable information, based on this study. Not on the “results” (line 303)	The generalizable information, based on this study  show healthy family functioning and hardiness in these families
P 15 L 321	
Line 303	check the grammar, the sentence seems not to be complete.	Thank hope you find it complete now	
SUBJECT	REVIEWER 2 COMMENTS	ANSWER	
Abstract	Where is the study period?	between December 2017 and June 2019. 
P2 L 32	
	Which type of software is used for analysis?	The data were coded and entered the Statistical Package for the Social Sciences (SPSS) version 25, for analysis. 
P2 L32-33	
	Regression results should be appeared in the result section	The data showed small variations between and within families for family functioning and family hardiness, there were only two families scoring low for both family functioning and hardiness. The variation was higher within the families, but there was no significance level.
P 2 L 39-42	
Introduction	What is the difference between introduction and background? Why you want to include both?	Background has been excluded in the text	
Method	Data collection tool and method	A quantitative cross-sectional design with validated self-rating scales was used to explore family functioning and family hardiness from patients' and family members' perspectives. P4 L 89-91	
	How many sample size you have?	Only one	
	How you select 60 families	The first author consecutively recruited the participants in collaboration with ICU administrators that listed from a database including former patients, participants eligible for inclusion.
P 5 L98-101	
	You used a consecutive sampling and recruiting 390 patients that fulfill the inclusion criteria and they receive the request to participate by means of an invitation letter but in in your abstract section you write 60 patients and 85 families with a total of 145 participants.	Using consecutive sampling, 390 participants fulfilling the inclusion criteria received a request to participate by means of an invitation letter, 60 answered and chose to participate.
P5 L 104-106	
Data analysis	How you calculate the power of your study? How it can be 104? How you calculate the power based on previous study? You should to calculate the power of your study?
Which type off software u used to calculate the power?	Power calculation based on data from a previous study using unpaired t-test. The calculation is based on a medium power size, 104 participants were needed.          ES = 0.6, á = 0:05; 1 − â = 0.8
P8 L169-171
The data were coded and entered the Statistical Package for the Social Sciences (SPSS) version 25, for analysis. L32-33	
Results	In total you include 145 participants. Is this from 390 patients or not please clarify it and provide the response rate?	Using consecutive sampling, 390 participants fulfilling the inclusion criteria received a request to participate by means of an invitation letter, 60 answered and chose to participate. 
P5 L 104-106
In total, data from 60 families (60 former ICU patients and their family members, 85; 145 participants)
P 9 L 206-207	
	In your data analysis section you write as you perform an inferential statistics (t-test and ANOVA) but I haven't seen any result of t-test and ANOVA?	See Table 1 and 2
