# Supplementary material for: Exploring family functioning and ‐ hardiness in families’ experiencing adult intensive care – A cross-sectional study
Source: PLoS One. 2023 Jul 6;18(7):e0288149. doi: 10.1371/journal.pone.0288149 (PMC10325063; doi:10.1371/journal.pone.0288149)
Supplement: Attachment — Submitted filename: Addressing the comments230523.docx [file pone.0288149.s003.docx]

Addressing the comments:

Thank you for reviewing this manuscript, the feedback of every subject was very clear and I hope you

| SUBJECT | JOURNAL REQUIREMENTS | ANSWER |
| --- | --- | --- |
| Reviewer #1 | Thank you very much for the revised version of the manuscript. All concerns have been addressed, and you submitted a very fine manuscript. Well done and thank you! | Thank you very much |
| Reviewer #2 | Thank you for considering my comments to strengthen your manuscript. | Thank you, I hope I have understood your comments and the manuscript have been improved with your constructive comments |
|  | 1. Better to include the software version you used in for analysis in the abstract section | I have included: version 25 on raw 33 |
|  | 2. Using consecutive sampling, you invited 390 participants. Then, 60 answered and chose to participate. In total, data from 60 families (60 former ICU patients and their family members, 85; 145 participants). The response rate will be 37.18%. How do you see it? Is it acceptable in research? | Included in Limitations, raw 294: Although power calculations show that medium size power was achieved, the low response rate might have led to an inaccurate positive picture. It seems resonable to presume that the most frail patients are not included in the study. Also of worth to consider is that low response rates produces bias only to the extent that there are differences between responders and non-responders on the estimate(s) of interest (37). Due to ethical considerations non-responders estimates are unknown in the study. |
|  | 3. Table 1 and 2 describes the descriptive statistics only. They do not show the inferential statistics (t-test and ANOVA) but I haven’t seen any result of t-test and ANOVA? You should do t-test and ANOVA analysis | The results of the t-test comparing the means (or averages) among ICU patients, family members and families to see if the groups differed, and the ANOVA exploring family functioning and family hardiness were non significant and are not reported.  Raw 209-211 |

find the adjustments to be appropriate.
